# Changes in health-related quality of life before and after a 12-month enhanced primary care model among chronically ill primary care patients in Australia

**DOI:** 10.1186/s12955-020-01539-1

**Published:** 2020-08-24

**Authors:** James Rufus John, W. Kathy Tannous, Amanda Jones

**Affiliations:** 1 Translational Health Research Institute, Western Sydney University, Campbelltown, Sydney, NSW 2560 Australia; 2 Rozetta Institute (formerly Capital Markets Cooperative Research Centre), The Rocks, Sydney, NSW 2000 Australia; 3 School of Business, Western Sydney University, Parramatta, NSW 2150 Australia; 4 Sonic Clinical Services, The Rocks, Sydney, NSW 2000 Australia

**Keywords:** Quality of life, EuroQol (EQ-5D), Multimorbidity, Chronic disease, Integrated care, Collaborative care, Chronic care model, Australia

## Abstract

**Purpose:**

Evidence suggests that Patient-centred Medical Home (PCMH) model facilitates person-centred care and improves health-related quality of life for patients with chronic illness. This study aims to evaluate changes in health-related quality of life (HRQoL), before and after enrolment into a 12-month integrated care program called ‘WellNet’.

**Methods:**

This study includes 616 eligible consented patients aged 40 years and above with one or more chronic conditions from six general practices across Sydney, Australia. The WellNet program included a team of general practitioners (GPs) and clinical coordinators (CCs) providing patient-tailored care plans configured to individual risk and complexity. HRQoL was recorded using the validated EuroQol five dimensions five levels (EQ-5D-5L) instrument at baseline and 12 months. Additionally, patients diagnosed with osteoarthritis also reported HRQoL using short versions of Knee and/or Hip disability and osteoarthritis outcome scores (KOOSjr and HOOSjr). A case-series study design with repeated measures analysis of covariance (ANCOVA) was used to assess changes in mean differences of EQ-5D index scores after controlling for baseline covariates. Additionally, backward stepwise multivariable linear regression models were conducted to determine significant predictors of EQ-5D index scores at follow-up.

**Results:**

Out of 616 patients, 417 (68%) reported EQ-5D scores at follow-up. Almost half (48%) of the WellNet patients reported improved EQ-5D index scores at follow-up. After controlling for baseline covariates, the adjusted mean difference was statistically significant whilst also meeting the bare minimal clinically important difference (MCID) with a change of 0.03 (95% CI 0.01, 0.05). The multivariable regression models determined that baseline EQ-5D scores and positive diagnosis of a respiratory illness were significant predictors of HRQoL at follow-up. There were significant improvements across both KOOS and HOOS assessments, specifically, the pain and symptom scores in both scales met statistical significance in addition to meeting the MCID.

**Conclusion:**

Patient-tailored chronic disease management (CDM) plans designed by team of GPs and CDM clinical coordinators could lead to better HRQoL among primary care patients.

## Introduction

The exponential rise in chronic disease prevalence presents significant public health burden to health care systems worldwide and challenges the need to revisit strategies towards effective prevention and management [1, 2]. In Australia, chronic conditions have accounted for 87% of deaths and a collective 61% of fatal and non-fatal burden in 2015 [3, 4]. Moreover, there is an increasing trend of multimorbidity among the ageing population resulting in greater demand for integration of health services [5, 6]. The health and economic ramifications of chronic illness in terms of premature mortality [7], polypharmacy [8], complexity of care [9], and diminished health-related quality of life [10] are well documented. Additionally, the increased health service utilisation among chronic disease sufferers is especially of interest, given Australia’s current fragmented health care framework which lacks continuity of care and care coordination [11, 12]. On the contrary, there is increasing evidence of improved health-related quality of life (HRQoL) in patients receiving collaborative and patient-centred care [13, 14].

In recent decades, there has been a paradigm shift in the measure of health care evaluation from the traditional health indicators of mortality and morbidity towards a broader perspective of patient reported outcome measures (PROMS) including daily functioning, quality of life, symptoms, and other aspects of their health [15, 16]. HRQoL is a multi-dimensional concept that measures the impact and quality of health encompassing an individual’s physical, mental, and social functioning [17, 18]. Determining the HRQoL for patients with chronic illness in primary care setting is beneficial as it enables understanding of patient’s insights and perception on where care needs to be directed in relation to their condition [19, 20]. This in turn allows providers to improve self-management behaviours among patients to effectively manage their conditions and symptoms [21]. There is evidence showing strong association between patient-provider communication and improved HRQoL [22, 23].

Australia has a long-standing use of surveys to measure population health quality and status, including recent incorporation of health utility measures. Data from the South Australia’s annual Health Omnibus Survey (HOS) and New South Wales’ 45 and Up Study have been extensively used to study HRQoL in several wide-level population norms [24, 25]. For instance, trends in the HRQoL study by Atlantis et al. shows that HRQoL significantly worsened over a 10-year period (1998–2008) for individuals with comorbid conditions compared to those with a single chronic condition [26]. Despite recent work, current knowledge of the HRQoL among specific population groups, like primary care patients with one or more chronic conditions, remains largely unknown. Therefore, the aim of this study is to evaluate the HRQoL, before and one-year after enrolment into an enhanced primary care program, and to investigate predictors of change in the HRQoL among primary care patients presenting with one or more chronic conditions.

## Methods

### WellNet program - overview, intervention, and study design

The ‘WellNet’ program developed by Sonic Clinical Services (SCS) is a general practitioner (GP) led, multidisciplinary team-based (MDT) care delivery model within primary care settings. The 12-month program is built upon best practice clinical care models, including the Patient-Centred Medical Home (PCMH), which aims to deliver care that is tailored to individual risk and comorbidity burden [27].

The enhanced primary care program is designed to provide individualised ‘whole-person’ care with focus on self-management support, health coaching and education, care coordination, shared decision making, and long-term continuity of care. Ongoing support and monitoring were provided through a total of 14 possible consultations with the care team in the form of in-practice visits and telephone contacts throughout the 12-month period. In addition, patients were also supported with a user-friendly online platform called ‘GoShare’ providing patient-tailored educational materials and a mobile application ‘MediTracker’ enabling access and reminders to the next scheduled GP appointments and prescriptions. Further details on how CCs monitored usage of GoShare and MediTracker are reported elsewhere [27].

Patients were recruited between December 2016 and October 2017 using a targeted convenience sampling technique if they met the eligibility criteria. Targeted convenience sampling is a commonly used non-probability sampling in clinical research where members of the target population that meet certain practical eligibility criteria are included for the purpose of the study [28]. A case-series study design was used to determine changes in the HRQoL before and after WellNet care among patients enrolled in six primary care practices across Northern Sydney, Australia. Informed consent was obtained from all participants upon enrolment into the WellNet program.

### Participants

**Fig. 1:**
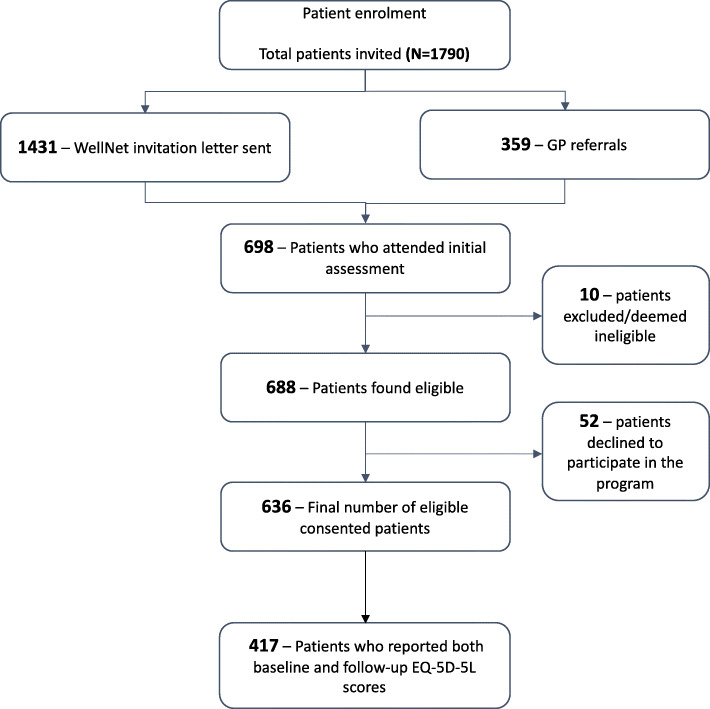
Flowchart of patient participation

A computerised algorithm was executed to identify potentially eligible patients from the electronic medical records of SCS GP practices. The overarching criteria for eligibility include patients aged 40 years and over; having one or more chronic condition/s with or without one or more elevated clinical risk factors; and had visited a GP at least thrice in the previous 2 years. Patients living in nursing homes and those with severe cognitive impairment or terminal illness (*n* = 10) were excluded. More details on the risk algorithm, enrolment, and data collection are reported elsewhere [27]. Of the 636 consenting participants, 616 who completed the EuroQol five dimensions and five levels version (EQ-5D-5L) questionnaire at baseline were analysed in this study. Flowchart of the enrolment outcomes is shown in Fig. 1.

### EQ-5D-5L instrument

The HRQoL was measured using the standardised UK version of the EQ-5D-5L instrument [29]. The questionnaire covers five dimensions of health: mobility, self-care, usual activities, pain or discomfort, and anxiety or depression. The levels of severity range from no problems (1) to extreme problems (5) for each of the five dimensions are recorded. The raw scores are then converted to a single EQ-5D index value using a scoring algorithm ranging from 0 (worst perceived health state) to 1 (best perceived health state) [30]. In this study, the UK version of scoring algorithm and value set were employed to calculate utility scores as an Australian scoring algorithm is unavailable for the 5 L version. The UK algorithm was estimated using a hybrid model of preference data collected using a time-trade off and discrete choice experiment methods [29, 30] and potential values from this algorithm ranged from − 0.281 to 1, where values lower than 0 represent states considered to be worse than death [29].

In the WellNet program, EQ-5D-5L questionnaire was recorded at baseline and 12 months (at program completion). For studies measuring the impact of treatment outcome/s, the minimal clinically important difference (MCID) reports on the smallest change in the outcome of interest that is considered to be clinically significant or meaningful [31]. A comprehensive review of 18 studies by Coretti et al. [32] estimated the overall MCID for EQ-5D-5L range to be, for musculoskeletal disorders, between 0.03 and 0.54.

### KOOS and HOOS assessments (short versions)

The Hip disability and Osteoarthritis Outcome Scores (HOOS) and Knee injury and Osteoarthritis Outcome Scores (KOOS) are shortened but validated versions of the HOOS and KOOS surveys indicated for patients with a positive diagnosis of osteoarthritis and reporting different forms of hip and knee disability [33, 34]. These surveys are intended for use over short and long-term intervals to assess patient-reported changes in the quality of life in terms of changes in the levels of function, symptoms, and pain induced by a particular treatment [33, 34].

Both the questionnaires contain subscales of items recording patient’s QoL in terms of pain, function, daily living, and stiffness. The raw scores for each subscale range from 0 to 24 which are then converted to interval scores using online calculators made available on the Ortho Tool kit website [35]. The interval scores range from 0 to 100: 0 indicates total disability, whilst 100 indicates perfect functionality. As MCIDs were not established at the start of this study, previous body of literature estimations were adopted where MCID values ranging between 9.6 and 16.2 for HOOS; and between 8 and 10 for KOOS [36, 37]. In this study, the HOOS and KOOS scales were only used as a supplement to EQ-5D instrument.

### Study outcomes and exploratory variables

The primary outcome of interest was changes in the EQ-5D index value and increase in the proportion of patients at ‘no problem’ level in all five dimensions at follow-up. The secondary outcomes included: 1) predictors of change in EQ-5D index over time; 2) adjusted mean difference in KOOS and HOOS scales recorded among subsample of patients diagnosed with osteoarthritis.

The explanatory or predictor variables analysed and adjusted for in this study as follows: age, gender, diagnosis of chronic conditions, number of co-existing conditions, private health insurance status (PHI), and number of scheduled consultations.

The changes in the levels of each dimension from baseline to follow-up were classified as follows: - - no problem level to no problem level (no change); − + lower problem level to a higher problem level (impairment); + − higher problem level to a lower problem level (improvement); + + higher problem to higher problem (no change).

### Data analysis

Descriptive statistics for continuous variables are expressed as mean and standard deviation (SD) whereas frequency counts of categorical variables are shown in percentages. Normality of distribution was assessed using the Shapiro-Wilk test for normality and by analysis of normal quantile-quantile plots. Independent samples t-tests and Pearson’s chi-square tests were conducted to determine significant differences between completers and those who withdrew (non-completers) before program completion. Additionally, Pearson’s product-moment correlation coefficient was conducted to determine the level of association between EQ. 5D scores and different chronic condition groups at baseline.

Unadjusted mean differences between baseline and follow-up were computed using paired samples t-test. The adjusted mean differences were determined by using the repeated measures analysis of covariance (ANCOVA) whilst adjusting for potential baseline covariates such as age, gender, diagnosis of chronic conditions, number of co-existing conditions, PHI status, and number of scheduled consultations. Additionally, subgroup analyses were also conducted to evaluate adjusted differences in EQ. 5D scores between proportion of patients based on number of chronic conditions (one and two or more conditions) and median contacts (< 12 contacts and ≥ 12 contacts) with WellNet care team.

To determine predictors of change in EQ-5D over time, multivariable linear regression models were employed using post-EQ. 5D index scores as outcome variable. Post-EQ. 5D index scores were preferred over change scores (follow-up minus baseline) as outcome variable because change scores fail to allow for optimal control of the baseline imbalance owing to potential regression to the mean [38, 39]. Univariate linear regression was conducted for each variable separately and variables with *p*-value< 0.20 were included in the multivariable model. The backward stepwise regression approach was used to reduce and create the final model while simultaneously assessing the fitness of model in order to avoid dropping of non-significant variables that may affect the model fitness. The final model constitutes variables, which when excluded, cause a prominent deviance change (*p* < 0.05) as compared to the corresponding X^2^ test statistic on the relevant degrees of freedom.

Finally, the internal consistency of EQ-5D, KOOS, and HOOS scales in this study were evaluated using Cronbach’s alpha. All analyses were conducted using SPSS (version 25) and R statistical software.

## Results

### Baseline characteristics and EQ-5D-5L scores

Baseline characteristics of patients including chronic disease prevalence, overall and stratified by completion status, are presented in Table 1. With exception to slight differences in proportions of those diagnosed with musculoskeletal disorder, no significant differences were observed between those who completed baseline and follow-up assessments and those completing baseline assessment only in terms of sociodemographic characteristics or clinical measures. Patients were on average 68.9 years old with almost similar gender distribution and had a mean number of 2 ± 1 chronic conditions. Diabetes (49%) was observed to be the most prevalent of the chronic conditions with cancer (14%) being the least prevalent among the WellNet patients. Additionally, more than two-thirds (69%) of the participating patients had private insurance.

**Table 1:** Baseline patient characteristics (Overall, by completion status)

Variable	Overall (***N*** = 616)	Baseline and follow-up assessments (***n*** = 417)	Baseline assessment only (***n*** = 199)	***p***-value
Age in years, Mean (SD)	68.9 (12.9)	69.6 (12.1)	67.4 (14.2)	0.063
**Gender**				
*Males*	306 (49.7)	200 (48.0)	106 (53.3)	0.218
*Females*	310 (50.3)	217 (52.0)	93 (46.7)	
**History of co-existing conditions**				
*Cardiovascular disease*	211 (34.3)	147 (35.3)	64 (32.2)	0.450
*Respiratory disease*	179 (29.1)	119 (28.5)	60 (30.2)	0.680
*Diabetes*	302 (49.0)	213 (51.1)	89 (44.7)	0.140
*Musculoskeletal disorders*	263 (42.7)	191 (45.8)	72 (36.2)	**0.024**
*Mental illness*	126 (20.5)	81 (19.4)	45 (22.6)	0.359
*Cancer*	89 (14.4)	57 (13.7)	32 (16.1)	0.426
**Number of co-existing conditions, Mean (SD)**	1.9 (0.9)	1.9 (1.0)	1.8 (0.9)	0.150
**Chronic conditions**				
One condition	219 (35.6)	141 (33.8)	78 (39.2)	0.192
Two or more conditions	397 (64.4)	276 (66.2)	121 (60.8)	
**Program contacts**				
< 12 contacts	313 (50.8)	130 (31.2)	183 (92.0)	**< 0.001**
≥ 12 contacts	303 (49.2)	287 (68.8)	16 (8.0)	
**Insurance status**				
*Private*	391 (68.5)	268 (68.9)	123 (67.6)	0.753
*Uninsured*	180 (31.5)	121 (31.1)	59 (32.4)	
**Mean EQ-5D-5L score**	0.79 (0.19)	0.79 (0.18)	0.78 (0.21)	0.493
**Clinical measures**				
*Systolic Blood Pressure (mmHg), Mean (SD)*	138.8 (19.2)	138.9 (18.6)	138.3 (20.3)	0.696
*Diastolic Blood Pressure (mmHg), Mean (SD)*	75.8 (18.0)	76.2 (17.1)	75.1 (19.9)	0.500
*Body Mass Index Kg/m2, Mean (SD)*	29.9 (7.3)	29.6 (6.4)	30.5 (8.7)	0.146
*Glycated Haemoglobin (%), Mean (SD)*	6.8 (1.4)	6.8 (1.5)	6.7 (1.3)	0.380
*High Density Lipoprotein Cholesterol (mmol/L), Mean (SD)*	1.3 (0.4)	1.3 (0.4)	1.3 (0.4)	0.695
*Low Density Lipoprotein Cholesterol (mmol/L), Mean (SD)*	2.7 (1.1)	2.7 (1.0)	2.9 (1.1)	0.059
*Total Cholesterol (mmol/L), Mean (SD)*	4.8 (1.4)	4.8 (1.3)	4.9 (1.5)	0.105
*Triglyceride (mmol/L), Mean (SD)*	1.7 (1.1)	1.6 (1.1)	1.7 (1.2)	0.352

Data presented as N (%) unless specified otherwise

Variables reported as percentages were tested with chi-square analyses and variables reported as means and standard deviations were tested with independent samples t-test

Bold letters suggest statistical significance with *p*-value< 0.05

**Fig. 2:**
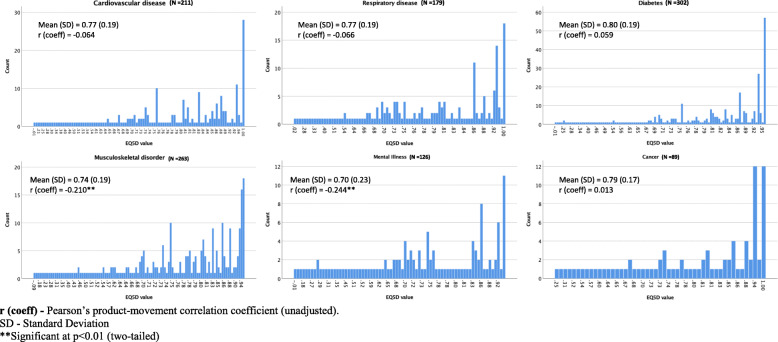
Distribution of baseline EQ. 5D index value by type of chronic condition

The overall mean (SD) EQ-5D index value of the sample at baseline was 0.79 (0.19). Of the 616 patients who reported their baseline EQ-5D scores, 91 (15%) patients reported ‘no problems’ across all five dimensions at baseline. 417 out 616 (68%) participants reported follow-up EQ-5D upon program completion. In terms of the type of chronic condition, people diagnosed with a mental illness reported the least mean (SD) EQ-5D index value of 0.70 (0.23) at baseline. Additionally, results of Pearson’s product-moment correlation coefficient (unadjusted) showed a small but statistically significant negative association between baseline EQ-5D index value and history of a mental illness (*r =* − 0.24, *p* < 0.001) and musculoskeletal disorder (*r =* − 0.21, *p <* 0.001) at baseline. The distribution of baseline EQ-5D index value by type of chronic conditions with Pearson’s correlation coefficients are presented in Fig. 2.

The internal consistency of EQ-5D items in this study was sound (Cronbach’s alpha coefficients = 0.85). Additionally, HOOS and KOOS items also showed high reliability with Cronbach’s alpha coefficients of 0.91 and 0.89 respectively.

### Changes in the EQ-5D-5L scores

The unadjusted within-group changes in the index value scores between baseline and follow-up showed statistically significant improvement with mean difference of 0.03 (95% CI 0.02, 0.05). After controlling for baseline covariates, the adjusted mean difference remained significant with 0.03 (95% CI 0.01, 0.05) (Table 2).

**Table 2:** Repeated measures ANCOVA with overall and subgroup analyses

Variable	Complete cases analysis (***N*** = 417)	
	Unadjusted mean difference (95% CI)	Adjusted mean difference (95% CI)^**a**^
Overall	0.03 (0.02, 0.05)**	0.03 (0.01, 0.05)**
**Subgroup analyses**		
**Number of chronic conditions**		
One chronic condition (*n* = 141)	0.01 (0.00, 0.03)	0.01 (−0.01, 0.03)
Two or more chronic conditions (*n =* 276)	0.04 (0.02, 0.06)**	0.04 (0.02, 0.06)*
**Median program contacts**		
< 12 contacts (*n =* 130)	0.03 (0.01, 0.06)*	0.03 (0.01, 0.07)*
≥ 12 contacts (n = 287)	0.03 (0.01, 0.05)**	0.03 (0.00, 0.04)*

^**a**^Adjusted for age, gender, diagnosis of chronic conditions, number of co-existing conditions, private insurance status (PHI), and number of scheduled consultations

**p-value< 0.001

*p-value< 0.05

Additionally, the subgroup analysis based on number of chronic conditions showed that patients with two or more chronic conditions (*N* = 276) observed higher adjusted mean difference in EQ. 5D scores of 0.04 (95% CI 0.02 to 0.06; *p*-value< 0.05) compared to patients with one chronic condition with adjusted mean difference of 0.01 (95% CI − 0.01 to 0.03; *p-*value = 0.258). However, patients who had 12 or more contacts (*N* = 287) with the care team had similar improvements in EQ. 5D scores as those with less than 12 contacts (*N* = 130) with adjusted mean differences of 0.03 (95% CI 0.00 to 0.04; *p-*value< 0.05) (Table 2).

In terms of the changes in the levels of each dimension from baseline to follow-up, the proportion of patients showing improvement from higher to lower problem level was higher in all dimensions compared to the proportion reporting impairment from lower to higher problem level at follow-up (Table 3).

**Table 3:** Changes in the levels of EQ. 5D by dimension between baseline and follow-up

EQ 5D dimensions	Change in the levels between baseline and follow-up	N (%)	***p***-value*
Mobility	No change (− −)	158 (37.9)	**< 0.001**
	Impairment (− +)	75 (18.0)	
	Improvement (+ −)	101 (24.2)	
	No change (+ +)	83 (19.9)	
Self-care	No change (− −)	335 (80.3)	**< 0.001**
	Impairment (− +)	25 (6.0)	
	Improvement (+ −)	46 (11.0)	
	No change (+ +)	11 (2.6)	
Usual activities	No change (− −)	172 (41.3)	**< 0.001**
	Impairment (− +)	65 (15.6)	
	Improvement (+ −)	116 (27.9)	
	No change (+ +)	63 (15.1)	
Pain/discomfort	No change (− −)	68 (16.3)	**< 0.001**
	Impairment (− +)	76 (18.2)	
	Improvement (+ −)	136 (32.6)	
	No change (+ +)	137 (32.9)	
Anxiety/Depression	No change (− −)	186 (44.6)	**< 0.001**
	Impairment (− +)	55 (13.2)	
	Improvement (+ −)	93 (22.3)	
	No change (+ +)	83 (19.9)	

- - no problem level to no problem level (no change); − + lower problem level to a higher problem level (impairment); + − higher problem level to a lower problem level (improvement); + + higher problem to higher problem (no change)

*p-values obtained from Monte Carlo method by crosstabulation of baseline vs follow-up levels

### Predictors of change in EQ-5D-5L scores over 12 months

Findings of the multivariable linear regression analyses showing significant predictors of EQ-5D scores at 12 months are presented in Table 4. Higher baseline EQ-5D-5L score was significantly positively associated with follow-up EQ-5D-5L scores (ß = 0.60; 95% CI 0.52 to 0.67 at *p* < 0.001). In addition, a positive diagnosis of respiratory disease was significantly negatively associated with EQ-5D-5L scores at 12 months compared to those without respiratory disease (ß = − 0.03; 85% CI − 0.06 to − 0.01 at *p* = 0.034). The number of chronic conditions was not significant at the final model.

**Table 4:** Multivariable linear regression analyses showing predictors of quality of life at 12-month follow-up using post EQ-5D-5L index value

Predictors	Complete cases analysis (***N*** = 417)	
	ß (95%CI)	***p***-value
Baseline EQ-5D-5L score	0.60 (0.52, 0.67)	< 0.001
Number of chronic conditions	0.02 (−0.01, 0.02)	0.832
Diagnosis of a respiratory disease		
No (*n* = 298)	1.00 (reference category)	
Yes (*n* = 119)	−0.03 (− 0.06, − 0.01)	0.034

ß – unstandardized beta coefficient (slope)

### Changes in KOOS and HOOS assessment at follow-up

Of the 97 patients with diagnosis of osteoarthritis, 55 reported KOOS outcomes and 30 reported HOOS outcomes at baseline and follow-up. There were significant improvements across all subscales of both the KOOS and HOOS assessments. Specifically, the pain and symptom scores in both scales met statistical significance in addition to meeting the MCID (Table 5). 24 out of 55 (44%) patients had adjusted mean differences met the MCID threshold for KOOS whereas 15 out of 30 (50%) patients had met the MCID criteria for HOOS at follow-up.

**Table 5:** Adjusted mean differences in HOOS and KOOS assessment (short version) at baseline and follow-up

Variables	Baseline Mean (SD)	Follow-up Mean (SD)	Adjusted mean difference^**a**^ (95% CI)	***P*** value
**KOOS scales (*N*** **= 55)**^**b**^				
KOOS pain score	65.0 (21.7)	76.2 (22.0)	10.6 (3.1, 18.2)	**0.05**
KOOS function score	62.5 (24.8)	69.8 (25.7)	6.5 (−1.6, 14.6)	0.114
KOOS symptom score	63.1 (17.8)	70.7 (18.7)	7.2 (1.2, 13.1)	**0.019**
KOOS stiffness score	61.8 (27.9)	68.2 (26.1)	7.0 (−1.1, 15.1)	0.090
**HOOS scales (*N*** **= 30)**^**c**^				
HOOS pain score	65.0 (23.1)	77.5 (25.1)	11.6 (3.2, 20.0)	**0.008**
HOOS function score	71.7 (17.8)	81.2 (20.4)	8.6 (1.1, 16.1)	**0.026**
HOOS symptom score	67.6 (16.6)	78.5 (19.6)	10.3 (3.7, 16.8)	**0.003**

^**a**^Adjusted for age, gender, diagnosis of chronic conditions, number of co-existing conditions, private insurance status (PHI), and number of scheduled consultations

^**b**^24 out of 55 (44%) patients had adjusted mean differences ≥ MCID for KOOS at follow-up

^c^15 out of 30 (50%) patients had adjusted mean differences ≥ MCID for HOOS at follow-up

The Pearson’s product-moment correlation coefficient correlation test between changes in EQ. 5D with HOOS and KOOS resulted in a weak but significant positive association of *r* = 0.395 and *r =* 0.285 respectively.

## Discussion

To our knowledge, WellNet study is the first study to evaluate the changes in HRQoL among patients with one or more chronic conditions in Australian primary care settings based on the principles of PCMH model. Findings of this study are consistent with the growing body of evidence showing strong association between patients’ HRQoL and several core elements of the PCMH such as involvement of a MDT [40, 41], continuity of care [42, 43], and shared decision making and patient-provider communication [22, 23]. Previous Australian studies by McCaffrey et al. [24] and others [44] have reported on health utilities and HRQoL on the general population norms using cross-sectional data. However, studies reporting on the disease-specific, high risk sub-group population using GP data are relatively less, which is of interest, as primary care is the forefront of care delivery in Australia with at least 85% of Australians consulting a GP every year [3]. In view of this, the WellNet study is novel as it closely examines the outcome of integrating care delivery on HRQoL at two different time points whilst determining predictors of change using GP data.

In this study, the use of EuroQol EQ-5D-5L over other instruments owes to its simplicity in accruing several aspects of an individual’s self-perceived health status in a relatively short duration through use of a short 5-item questionnaire [45]. Moreover, the EQ-5D-5L has also been reported as one of the sensitive instruments in terms of better discriminative power in effectively detecting changes in the HRQoL [46]. In addition, it is reported to have better known-group validity where subjective patient scores are shown to be in accordance with the objective investigator findings of changes in the HRQoL [47].

Findings of our study showed both statistical significance whilst also meeting the bare minimal threshold of clinical significance in EQ-5D index scores after adjusting for baseline covariates. However, considering that our sample is chronically ill with many patients having multiple diseases, MCID may not even be a significant indicator on population level. In this population, we would typically expect that many patients would have progressed in their disease, so even small change or no change in the EQ. 5D scale may be a positive outcome for the program. The effectiveness of PCMH model on improving patients’ HRQoL is consistent with studies by Schuttner et al. [13] and Hynes et al. [14].

Of the five dimensions of EQ-5D, WellNet patients reported substantial improvement particularly on two domains of pain/discomfort and usual activities in terms of a 33 and 28% increase, respectively, in the ‘no problem’ level at follow-up. This could be attributed to the primary objective of the WellNet program in improving self-management behaviour among patients to effectively manage symptoms associated with their chronic conditions [27]. Improved self-management behaviours are strongly associated with improved HRQoL [48, 49].

Findings of the multivariable regression model show that higher baseline index value and positive diagnosis of respiratory disease were significantly associated with EQ-5D index at 12 months. Higher baseline EQ-5D index value as significant predictor of increased follow-up index scores is consistent with other study findings by Van Eck et al. [39]. This could be because patients who already reported better HRQoL at baseline benefitted through further patient education and self-management from the WellNet care team. A positive history of respiratory disease was negatively associated with HRQoL at follow-up compared to those without prior respiratory disease. The poor HRQoL reported among patients with respiratory disease due to several reasons of duration and severity of the condition supplemented with or without harmful lifestyle behaviours is well documented [50, 51].

KOOS and HOOS assessments were recorded in parallel with EQ. 5D instrument in the WellNet study. Changes in the KOOS and HOOS scores were supplemented with the primary outcome of EQ. 5D changes. Besides statistical significance, the scores also met the MCID rendering them clinically relevant for changes in the patient management. The favourable changes in this study is consistent with findings of other studies of collaborative care [52, 53].

Our study has several strengths and limitations. This is the first study in Australia to evaluate the outcome of a PCMH model on HRQoL among patients in primary care setting. The study includes an effectively targeted sample with longitudinal measurements at two different time intervals enabling determining predictors of change in the HRQoL scores. This study also exhibited a good response rate of 68% which is high and satisfactory in this kind of survey [54]. This study also adds to the relatively less than adequate research conducted using GP-data. Although the aim of this study was to evaluate changes in HRQoL after the 12-month WellNet intervention, this study was not designed as an effectiveness study, but rather as a proof-of-concept study.

In regard to study limitations, although WellNet program comprises an effectively matched comparison group, the EuroQol EQ-5D-5L was recorded only among treatment group, thereby limiting to within-group analysis. The lack of control group means that the possibility of potential bias cannot be excluded, and we cannot be sure that improvement in EQ. 5D scores may have occurred anyway without the enhanced PCMH intervention. However, that seems unlikely based on research conducted with use of control groups reporting similar outcomes [55, 56]. Additionally, some key socio-economic variables such as annual income were unavailable due to privacy concerns, which may also have impacted prediction of the index scores over time. With exception of HOOS and KOOS surveys, we did not have other validated instruments to determine HRQoL in parallel with the EQ-5D-5L instrument, which could have further increased the reliability of the findings. Finally, consistent with other originally designed programs, reproducibility of findings is constrained by potential barriers in the form of uniqueness of data and by patient and provider-level determinants [27, 57].

## Conclusion

Evaluating the HRQoL for patients with chronic illness enables understanding of patient’s insights and perception on where care needs to be directed in relation to their condition. The integration of GPs and trained CDM coordinators proves critical for provision of individualised care for patients presenting with multiple chronic conditions. This study demonstrates outcome of integrating care delivery on HRQoL at two different time points whilst determining predictors of change using GP data. Besides statistical significance, patients also met the MCID rendering them clinically relevant for change in patient management. Future research should seek to evaluate the sustained effects and cost-benefits of the WellNet program.

## Data Availability

WellNet data may not be available to the general public for security reasons.

## Abbreviations

ANCOVA: Analysis of covariance
BMI: Body mass index
CC: Clinical coordinator
CDM: Chronic disease management
CI: Confidence interval
CVD: Cardiovascular disease
EQ-5D-5L: EuroQol Five Dimensions Five Levels
FCS: Fully conditional specification
GP: General practitioner
HOS: Health Omnibus Survey
HOOS: Hip disability and osteoarthritis outcome scores
HRQoL: Health-related quality of life
KOOS: Knee injury and Osteoarthritis Outcome Scores
MCID: Minimal clinically important difference
MCMC: Markov Chain Monte Carlo
MDT: Multidisciplinary team
NSW: New South Wales
PCMH: Patient Centred Medical Home
PHI: Private health insurance
PROMS: Patient reported outcome measures
SCS: Sonic Clinical Services
SD: Standard Deviation
SPSS: Statistical Package for the Social Sciences
